# Transcriptomic and alternative splicing analyses provide insights into the roles of exogenous salicylic acid ameliorating waxy maize seedling growth under heat stress

**DOI:** 10.1186/s12870-022-03822-3

**Published:** 2022-09-09

**Authors:** Jian Guo, Zitao Wang, Lingling Qu, Yifan Hu, Dalei Lu

**Affiliations:** 1 Jiangsu Key Laboratory of Crop Genetics and Physiology/Jiangsu Key Laboratory of Crop Cultivation and Physiology/Agricultural College of Yangzhou University, Yangzhou, 225009 People’s Republic of China; 2 Jiangsu Co-Innovation Center for Modern Production Technology of Grain Crops, Yangzhou University, Yangzhou, 225009 People’s Republic of China; 3 Joint International Research Laboratory of Agriculture and Agri-Product Safety of the Ministry of Education, Yangzhou University, Yangzhou, 225009 People’s Republic of China

**Keywords:** Waxy maize, Seedling, Heat stress, Salicylic acid, Transcriptome

## Abstract

**Background:**

Salicylic acid (SA) is a phytohormone which works to regulate the abiotic stress response of plants. However, the molecular mechanism by which SA mediates heat tolerance in waxy maize (*Zea mays* L. *sinsensis* Kulesh) remains unknown.

**Results:**

Two varieties of waxy maize seedlings, heat-tolerant ‘Yunuo7’ (Y7) and heat-sensitive ‘Suyunuo5’ (S5), were pretreated with SA prior to heat stress (HTS). After treatment, physiological and transcriptomic changes were analyzed. Compared with HTS, the exogenous application of SA enhanced the shoot dry weight, the activities of antioxidant enzymes (e.g., SOD, POD, CAT and APX), and the concentration of endogenous phytohormones (e.g., SA, ABA, IAA, GA3), while decreased the MDA content. Transcriptome analysis showed that the number of differentially expressed genes (DEGs) identified in the control (CK) vs HTS and HTS vs HTS + SA comparisons were more in S5 than in Y7. HTS induced the downregulation of genes involved in photosynthesis and the upregulation of genes encoding heat shock transcription factors (HSFs) and heat shock proteins (HSPs). Compared with HTS, SA pretreatment reversed the expression of 5 photosynthesis-related genes, 26 phytohormone-related genes, and all genes encoding HSFs and HSPs in S5. Furthermore, the number of alternative splicing (AS) events increased under HTS treatment for both varieties, while decreased under SA pretreatment of S5. Differentially spliced genes (DSGs) showed little overlap with DEGs, and DEGs and DSGs differed significantly in functional enrichment.

**Conclusions:**

Physiological and transcriptional together indicated that HTS and SA pretreatment had a greater effect on S5 than Y7. Additionally, it appears that transcriptional regulation and AS work synergistically to enhance thermotolerance in heat-sensitive waxy maize. Our study revealed the regulatory effects and underlying molecular mechanisms of SA on waxy maize seedling under HTS.

**Supplementary Information:**

The online version contains supplementary material available at 10.1186/s12870-022-03822-3.

## Background

Maize (*Zea mays* L.) is one of the most important crops grown worldwide, and is used as a food source for humans and animals, and as a raw material for many industrial purposes. Unfortunately, due to ongoing climate change, heat stress (HTS) has become one of the most severe abiotic threats to maize production [1, 2]. HTS during the growing season unbalances plant homeostasis, limiting growth and development and reducing crop yields [3–6]. In maize, the seedling stage is especially sensitive to HTS, resulting in chlorosis, stunting, and eventual death in severe cases [7, 8]. Further, HTS disrupts the endogenous hormone balance, promotes the overproduction of reactive oxygen species (ROS) and leadings to the oxidative damage of cell membranes and photosynthetic machinery [5, 9]. Because the global ambient temperature is projected to continue to rise during the next several decades [10], improving heat tolerance in maize is important to evade HTS-induced yield losses.

Salicylic acid (SA), a phenolic compound widely found in plants, is involved in the regulation of defense responses [11]. Previous studies have confirmed that SA is a safe plant protector and growth regulator [12]. The exogenous application of SA has been found to induce systemic resistance to HTS in different plant species, including alfalfa [13], rice [14], wheat [15], and maize [16]. SA acts to alleviate the oxidative damage induced by HTS by regulating the expression of heat-stable genes and proteins (e.g., *HSFs*, *HSPs*, *CDPK*, *SOD*, and *RCA*), ascorbate–glutathione cycle, ROS accumulation, and antioxidant enzyme activities [17–19]. Furthermore, SA accumulation in plant tissues activates the expression of defense-related genes, thereby participating in the regulation of heat tolerance [20]. Meanwhile, feedback between SA and other phytohormones modulates the co-regulation of the HTS response [21, 22]. A plant hormone signaling networks proposed by Verma et al. [23] described a series of SA-affected genes involved in regulating the synthesis and signal transduction of other phytohormones. In light of the present and future threat of HTS to maize production, it is thus of great importance to examine the mechanism by which exogenous SA confers heat tolerance in this crop.

Transcriptomic sequencing is a powerful molecular biological technique for studying the complex gene regulatory networks controlling plant adaptation and stress resistance to stress [24]. Specifically, transcriptomics has allowed us to systematically identify the genes and transduction pathways by which plants effectively respond to stress [25–27]. One important post-transcriptional regulatory mechanism which works to modulate the plant response to abiotic stress is alternative splicing (AS) of RNA [28]. AS refers to the generation of multiple transcripts from the same gene, each encoding a different functional protein [29]. In plants, and AS stimulates specific molecular functions and acts as a transcriptomic adaptation mechanism in response to abiotic stress [30, 31]. The occurrence of AS in stress-responsive genes is upregulated in response to HTS, thus affecting plant tolerance to HTS [32, 33].

Waxy maize, which kernel contains nearly pure amylopectin, is a popular food and industrial feedstock in many countries [34]. Summer waxy maize, grown primarily in China’s Huanghuaihai maize belt, is sown in May and June after harvesting wheat and canola [35]. However, in this region, exposure to HTS often occurs in June and July, often severely threatening the growth and development of waxy maize seedlings [36]. Little is known about how the application of SA affects gene expression and AS in maize in response to HTS. To address this knowledge gap, we pretreated two varieties of waxy maize seedlings with SA prior to HTS treatment. Next, seedlings were analyzed for physiological and transcriptomic changes. We found that SA pretreatment protects waxy maize seedlings through regulation of HTS-responsive gene expression and AS, antioxidant homeostasis, and phytohormone balance. Our results help to elucidate the mechanism of SA-mediated HTS tolerance in maize.

## Results

### Effect of SA on waxy maize growth and antioxidant homeostasis after HTS treatment

**Fig. 1:**
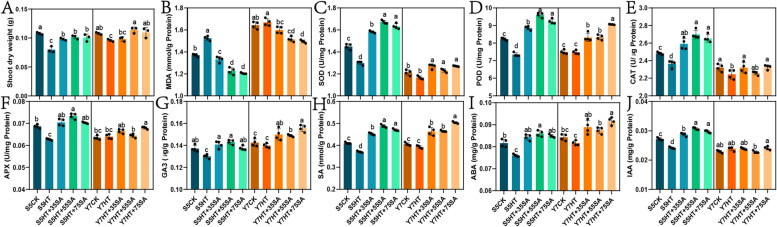
Effect of heat stress and SA on the physiological characteristics of waxy maize seedlings. **A**, shoot dry weight; **B**, MDA (malondialdehyde); **C**, SOD (superoxide dismutase); **D**, POD (peroxidase); **E**, CAT (catalase); **F**, APX (ascorbate peroxidase); **G**, GA3 (gibberellic acid); **H**, SA (salicylic acid); **I**, ABA (abscisic acid); **J**, IAA (indole-3-acetic acid). Different letters of the same variety under different treatments indicate significant difference at *P* < 0.05

Compared with the control, a decrease in shoot dry weight of 27.78 and 11.11% were observed in S5 and Y7 under HTS treatment, respectively (Fig. 1A). Treatment of S5 with 0.35, 0.55 and 0.75 mM of SA and Y7 with 0.55 and 0.75 mM of SA resulted in a significant increase in shoot dry weight (*P* < 0.05, Fig. 1A). After HTS treatment, S5 showed a significant increase in malondialdehyde (MDA) content, but not in Y7 (*P* < 0.05, Fig. 1B). In both varieties, the content of MDA decreased as exogenous SA concentration increased (Fig. 1B). In comparison to the control, HTS treatment significantly decreased the activities of superoxide dismutase (SOD), peroxidase (POD), catalase (CAT), and ascorbate peroxidase (APX) in S5 (*P* < 0.05), but not in Y7 (Figs. 1C, D, E, and F). In both varieties, the activities of these four antioxidant enzymes increased after application of exogenous SA. Among SA treatment, 0.55 mM SA had the greatest impact on the four antioxidant enzyme activities of S5, and 0.75 mM SA had the greatest impact on the POD and APX enzymatic activities of Y7.

### Effect of SA on waxy maize phytohormone balance after HTS treatment

Compared with the control, HTS resulted in a significantly decrease in the content of endogenous abscisic acid (ABA), indole-3-acetic acid (IAA), SA, and gibberellic acid (GA3) in S5 (*P* < 0.05), but not in Y7 (Figs. 1G-J). In both varieties, the contents of ABA, SA, and GA3 increased after application of exogenous SA. Additionally, the content of IAA increased after application of exogenous SA in S5, but not in Y7. After HTS treatment, 0.55 and 0.75 mM SA had the most pronounced effect on the phytohormone content in S5 and Y7, respectively.

### Transcriptomic analysis of waxy maize leaves under HTS and SA

Based on physiological data analysis, we selected leaves that are treated with CK, HTS, and HTS + 55SA (i.e., HTS + SA) of S5 and CK, HTS, and HTS + 75SA (i.e., HTS + SA) of Y7 for transcriptome sequencing (RNA-seq). RNA-seq generated an average of 21.09 million clean reads (range: 19.19–23.96) from each sample. At least 87.01% of the clean reads were mapped to the maize B73 reference genome (Gen_v4). For GC content, the Q30 and Q20 values ranged from 53.16–55.13%, 93.00–93.97%, and 97.38–97.99%, respectively (Table S1). Both the number of transcripts identified per sample (fragments per kilobase million, FPKM) and the FPKM correlation among the three biological replicates of each treatment indicated high quality and reproducibility (Fig. S1A). Across the 18 samples tested, ~ 46.46% of genes were not expressed, ~ 19.22% of genes were expressed at low levels (0 < FPKM < 1), ~ 31.93% of genes were expressed at moderate levels (1 ≤ FPKM < 60), and ~ 2.31% of genes were expressed at high levels (FPKM ≥60) (Fig. S1B). Principal component analysis (PCA) of FPKM values resulted in a clear separation among the three S5 treatments, and between CK and the two other Y7 treatments, but not between the HTS and HTS + SA for Y7 (Figs. S1 C and D).

### Identification and functional classification of differentially expressed genes (DEGs)

**Fig. 2:**
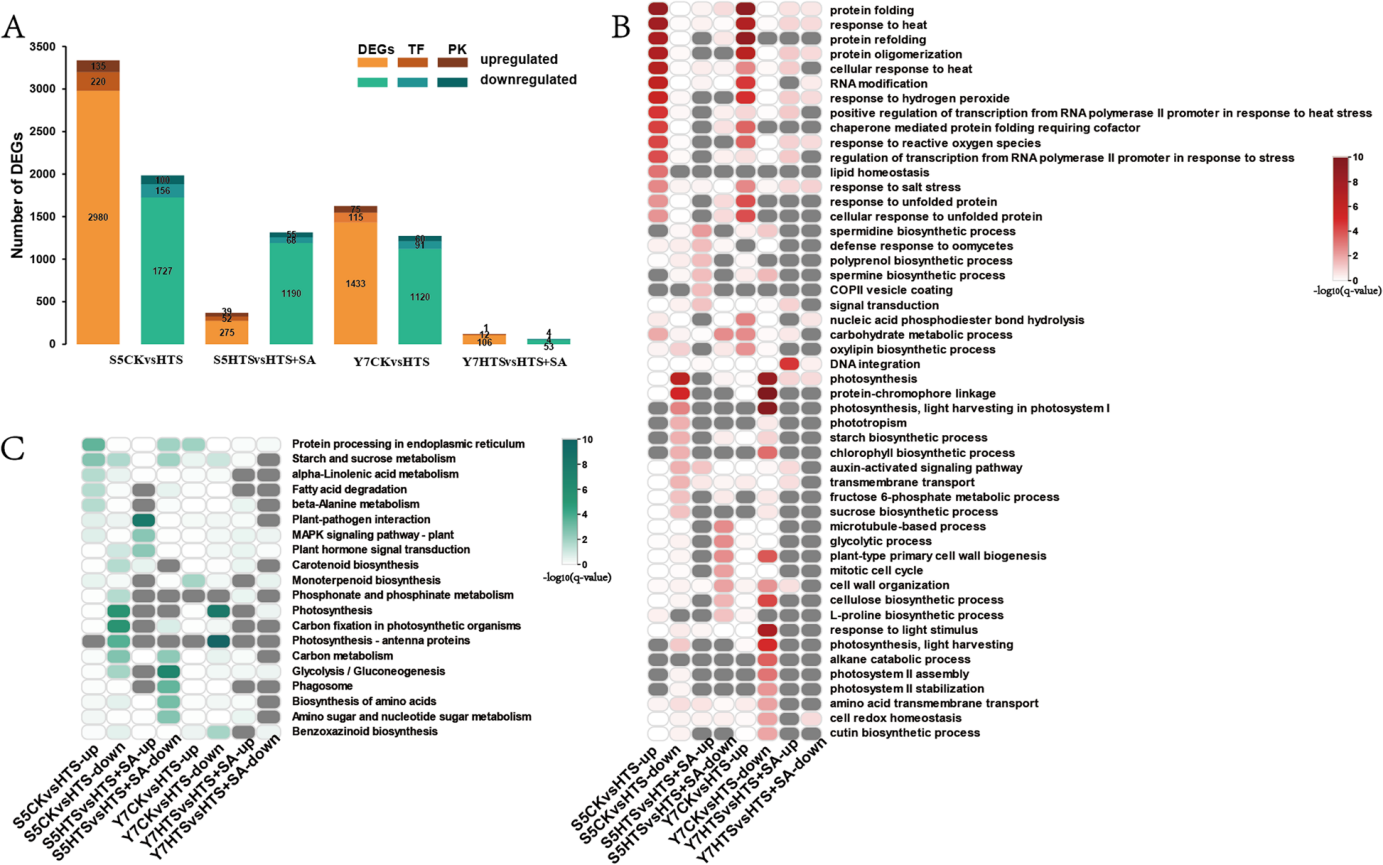
Effect of heat stress and SA on the transcriptome profiles of waxy maize seedlings. **A** numbers of differentially expressed genes (DEGs), TF-coded DEGs, and PK-coded DEGs in two comparisons of two varieties. **B** GO terms enrichment analysis of up- and downregulated genes in CK vs HTS and HTS vs HTS + SA comparisons of two varieties. **C** KEGG pathway analysis of up- and downregulated genes in CK vs HTS and HTS vs HTS + SA comparisons of two varieties. The color scale on the right of B and C represents the -log_10_ (*q*-value)

We identified 5318 DEGs (3335 upregulated and 1983 downregulated) in S5CK vs HTS, 1679 DEGs (366 upregulated and 1313 downregulated) in S5HTS vs HTS + SA, 2894 DEGs (1623 upregulated and 1271 downregulated) in Y7CK vs HTS, and 180 DEGs (119 upregulated and 61 downregulated) DEGs in Y7HTS vs HTS + SA (Fig. 2A). Among these DEGs, transcription factors (TFs) and protein kinases (PKs) ranged within 7.07–8.89% and 2.78–5.60% in different comparison groups, respectively. After SA pretreatment, S5 showed a greater number of DEGs than Y7, suggesting that the effects of exogenous SA on waxy maize seedlings under HTS may vary across varieties. To validate our RNA-seq data, we randomly selected eight DEGs and tested their transcript levels by qRT-PCR. Correlation analysis showed that the qRT-PCR results for these selected genes were highly correlated with RNA-seq data (R^2^ > 0.75, Fig. S2).

Gene ontology (GO) analysis indicated the upregulated DEGs in S5CK vs HTS and Y7CK vs HTS were enriched in protein folding, response to heat, protein refolding, protein oligomerization, and cellular response to heat (Fig. 2B). Downregulated DEGs were enriched in photosynthesis, protein–chromophore linkage, light harvesting, photosystem I, and chlorophyll biosynthetic process (Fig. 2B). In S5HTS vs HTS + SA, upregulated DEGs were enriched in spermidine biosynthetic process, defense response to oomycetes, and polyprenol biosynthetic process, while downregulated DEGs were enriched in microtubule–based process, glycolytic process, and plant–type primary cell wall biogenesis. In Y7HTS vs HTS + SA, upregulated DEGs were enriched only in DNA integration, while no GO term were enriched in downregulated DEGs (Fig. 2B).

Kyoto encyclopedia of genes and genomics (KEGG) pathway analysis indicated that the upregulated DEGs in S5CK vs HTS were enriched in protein processing in endoplasmic reticulum, starch and sucrose metabolism and alpha–linolenic acid metabolism, while downregulated DEGs were enriched in photosynthesis, carbon fixation in photosynthetic organisms, and photosynthesis–antenna proteins (Fig. 2C). In S5HTS vs HTS + SA, upregulated DEGs were enriched in plant–pathogen interaction, MAPK signaling pathway–plant, and plant hormone signal transduction, while downregulated DEGs were enriched in glycolysis/gluconeogenesis and phagosome. In Y7CK vs HTS, upregulated DEGs were enriched in protein processing in endoplasmic reticulum, while downregulated DEGs were enriched in photosynthesis–antenna proteins (Fig. 2C). In Y7HTS vs HTS + SA, upregulated DEGs was enriched in DNA integration, and no pathway were enriched in downregulated DEGs.

### Examination of DEGs related to plant hormone signal transduction

**Fig. 3:**
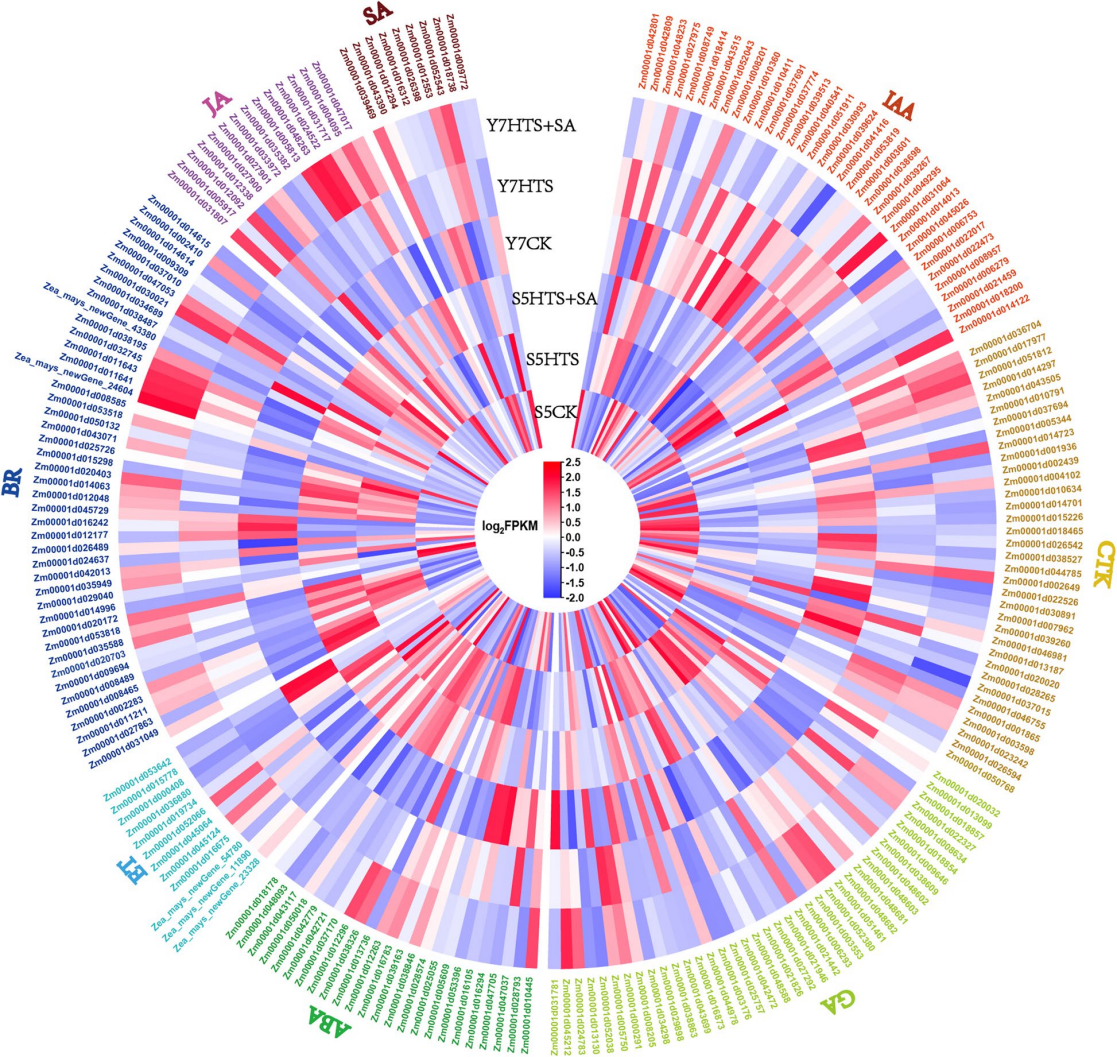
Expression analysis of genes related to plant hormone signal transduction. IAA, indole-3-acetic acid; GA, gibberellic acid; ABA, abscisic acid; BR, brassinosteroid; JA, jasmonic acid; SA, salicylic acid. Expression values of genes are presented as FPKM-normalized log_2_-transformed counts

Our analysis yielded 211 genes involved in plant hormone signal transduction pathways, including specifically the IAA, cytokinine (CTK), GA3, ABA, ethylene (ET), brassinosteroid (BR), jasmonic acid (JA), and SA (Fig. 3, Table S2). For the IAA pathway, 25 DEGs (8 upregulated and 17 downregulated) were found in S5CK vs HTS, and 9 DEGs (3 upregulated and 6 downregulated) were found in Y7CK vs HTS, and 8 DEGs were found in S5HTS vs HTS + SA, with expression patterns opposite that of S5CK vs HTS. For the GA3 pathway, 23 DEGs (15 upregulated and 8 downregulated) were found in S5CK vs HTS, 15 DEGs (9 upregulated and 6 downregulated) were found in Y7CK vs HTS, and 5 DEGs were found in S5HTS vs HTS + SA, with expression patterns opposite that of S5CK vs HTS. For the JA pathway, 7 DEGs (3 upregulated and 4 downregulated) were found in S5CK vs HTS, and 6 DEGs (4 upregulated and 2 downregulated) were found in Y7CK vs HTS, 4 DEGs were found in S5HTS vs HTS + SA, with expression patterns opposite that of S5CK vs HTS, and 2 DEGs were found in Y7HTS vs HTS + SA, with expression patterns opposite that of Y7CK vs HTS. For the CTK pathway, 22 DEGs (4 upregulated and 18 downregulated) were found in S5CK vs HTS, 11 DEGs (2 upregulated and 9 downregulated) were found in Y7CK vs HTS. For the SA pathway, in both S5 and Y7 CK vs HTS, 6 DEGs (2 upregulated and 4 downregulated) were found, and 2 DEGs were found in S5HTS vs HTS + SA, with expression patterns opposite that of S5CK vs HTS. For the ABA, ET and BR pathways, the number of upregulated genes was higher than that of downregulated genes in S5CK vs HTS, while the opposite was true in Y7CK vs HTS. Taken together, these results indicate that plant hormone signal transduction is affected under HTS, and the changes are relieved to some extent by application of exogenous SA, especially in S5.

### Examination of DEGs related to photosynthesis, heat shock transcription factors (HSFs), and heat shock proteins (HSPs)

**Fig. 4:**
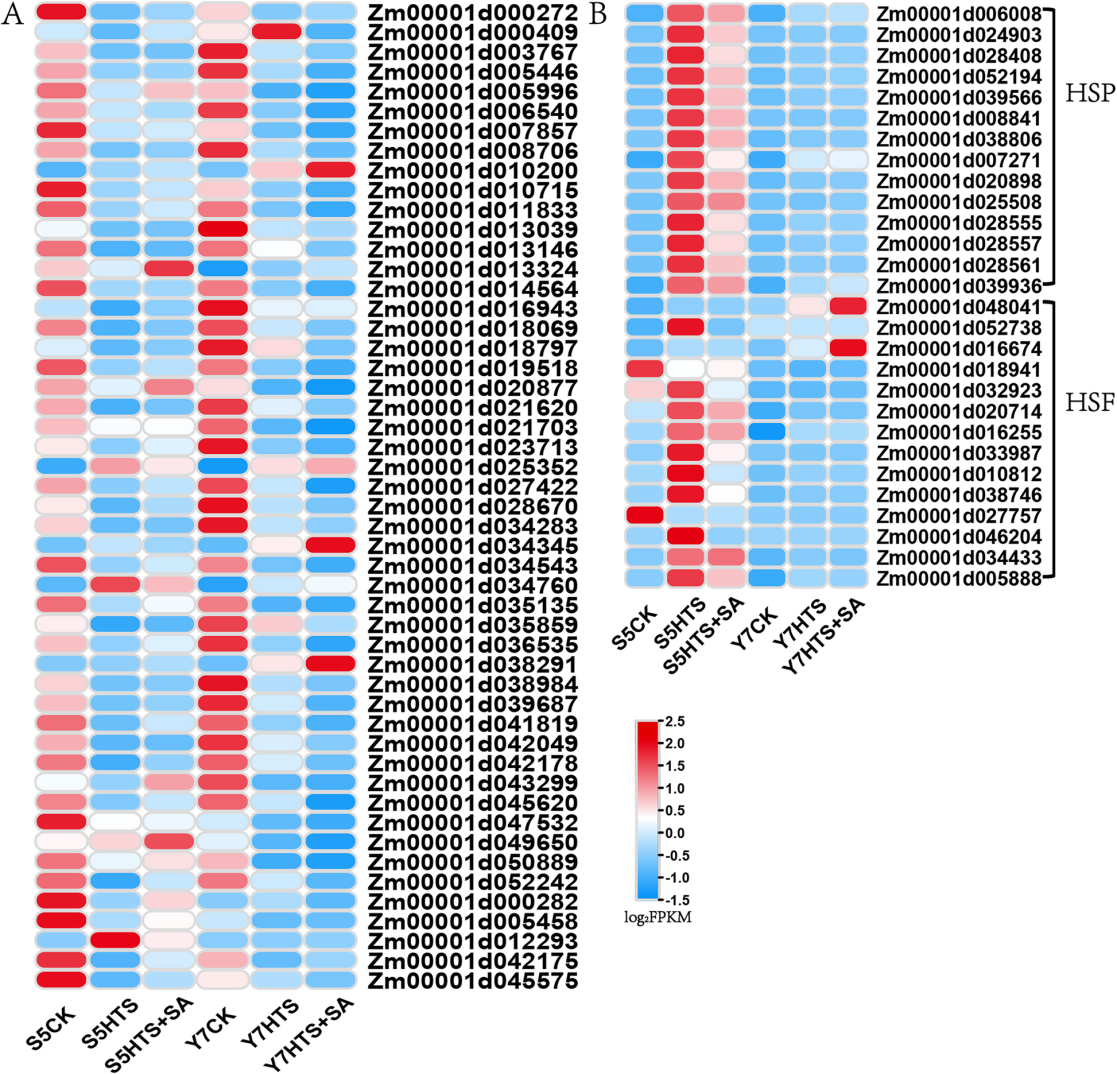
Expression analysis of genes related to photosynthesis, heat shock transcription factors and heat shock proteins. **A**, DEGs involved in photosynthesis pathway. **B**, DEGs encoded heat shock transcription factors (HSFs) and heat shock proteins (HSPs). Expression values of genes are presented as FPKM-normalized log_2_-transformed counts

Overall, it appears that HTS has a significant impact on leaf-level photosynthesis of both varieties (Fig. 4). In S5 seedlings, 21 photosynthesis-related genes were downregulated after HTS treatment, while 26 genes were downregulated in Y7 (Fig. 4A). In the comparison of S5HTS vs HTS + SA, 5 genes (e.g., Zm00001d000409, Zm00001d013324, Zm00001d043299, Zm00001d000282, and Zm00001d042175) were upregulated. Notably, exogenous application of SA did not alter the expression of photosynthesis-related genes in Y7 under HTS. Furthermore, 12 genes encoding HSFs and 14 genes encoding HSPs in S5 were induced to be upregulated by HTS treatment, and SA treatment decreased the expression of these genes (Fig. 4B). We also found that 4 genes encoding HSFs and 14 genes encoding HSPs in Y7 were upregulated under HTS treatment, and SA treatment had no effect on their expression. Compared with Y7, S5 activated more HSFs under HTS treatment, and the regulation effect of SA on S5 was more obvious.

### Identification and characterization of AS events

**Fig. 5:**
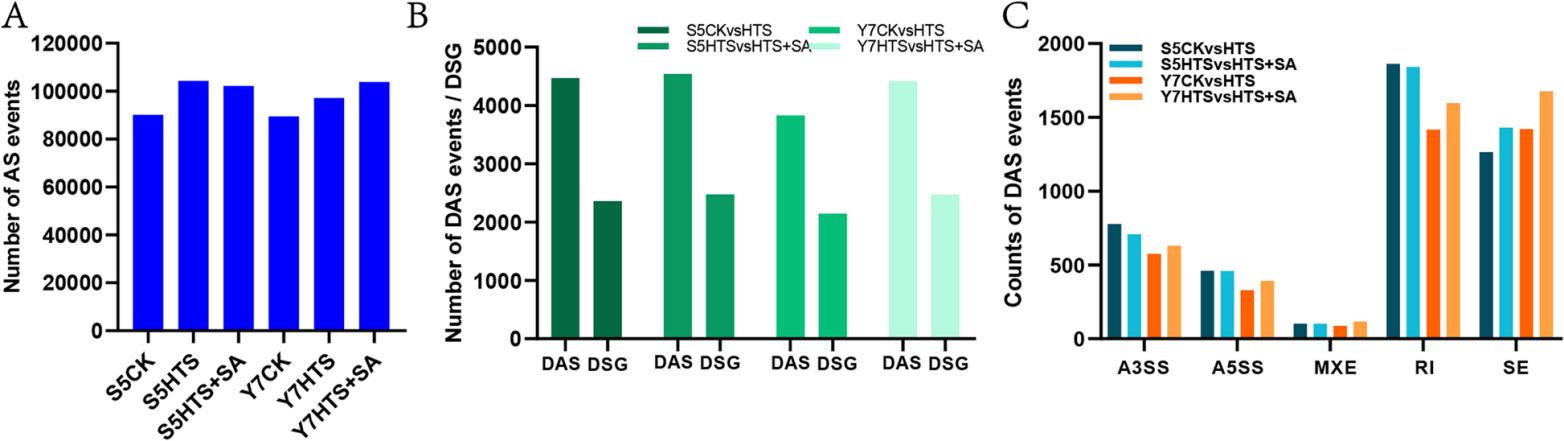
Effect of heat stress and SA on the alternative splicing events of waxy maize seedlings. **A**, Numbers of alternative splicing (AS) events in the leaves of two varieties under CK, HTS, and HTS + SA treatments. **B**, Numbers of differentially alternative splicing (DAS) events and corresponding genes (DSGs). **C**, The distribution of different type of AS events

A total of 90,181, 104,371, 102,180, 89,460, 97,193, and 103,810 AS events were identified in S5CK, S5HTS, S5HTS + SA, Y7CK, Y7HTS, and Y7HTS + SA, respectively (Fig. 5A). On this basis, 4467, 4540, 3831, and 4414 differentially alternative splicing (DAS) events were identified in S5CK vs HTS, S5HTS vs HTS + SA, Y7CK vs HTS, and Y7HTS vs HTS + SA, which correspond to 2359, 2475, 2146, and 2472 differentially spliced genes (DSGs), respectively (Fig. 5B). These DAS events were divided into 5 types, namely, retained intron (RI), skipped exon (SE), mutually exclusive exons (MXE), alternative 5’ splice site (A5SS), and alternative 3′ splice site (A3SS), of which RI and SE accounted for the largest proportion (Fig. 5C).

Both GO and KEGG enrichment analyses were performed on these DSGs. GO terms related to vesicle transport along actin filament, regulation of alternative mRNA splicing, via spliceosome, peptidyl–serine phosphorylation, and protein polyubiquitination were enriched in both S5CK vs HTS and S5HTS vs HTS + SA comparsions (Fig. S2). Two specific GO terms were enriched in S5CK vs HTS, namely, mRNA cis splicing, via spliceosome, and chromatin remodeling. In the comparison of Y7CK vs HTS, GO terms related to protein deubiquitination, and regulation of mitotic cell cycle were enriched, while in Y7HTS vs HTS + SA, only protein deubiquitination was enriched. In the comparison of S5CK vs HTS, 6 KEGG pathways were enriched, namely, mRNA surveillance pathway, ubiquitin mediated proteolysis, inositol phosphate metabolism, basal transcription factors, phosphatidylinositol signaling system, and glycerophospholipid metabolism. In the comparison of S5HTS vs HTS + SA, 3 KEGG pathways were enriched, namely, mRNA surveillance pathway, ubiquitin mediated proteolysis, and basal transcription factors (Fig. S3). Only 1 KEGG pathway was enriched in Y7CK vs HTS (mRNA surveillance pathway), and no KEGG pathways were enriched in Y7HTS vs HTS + SA.

### Comparative analysis of DEGs and DSGs

**Fig. 6:**
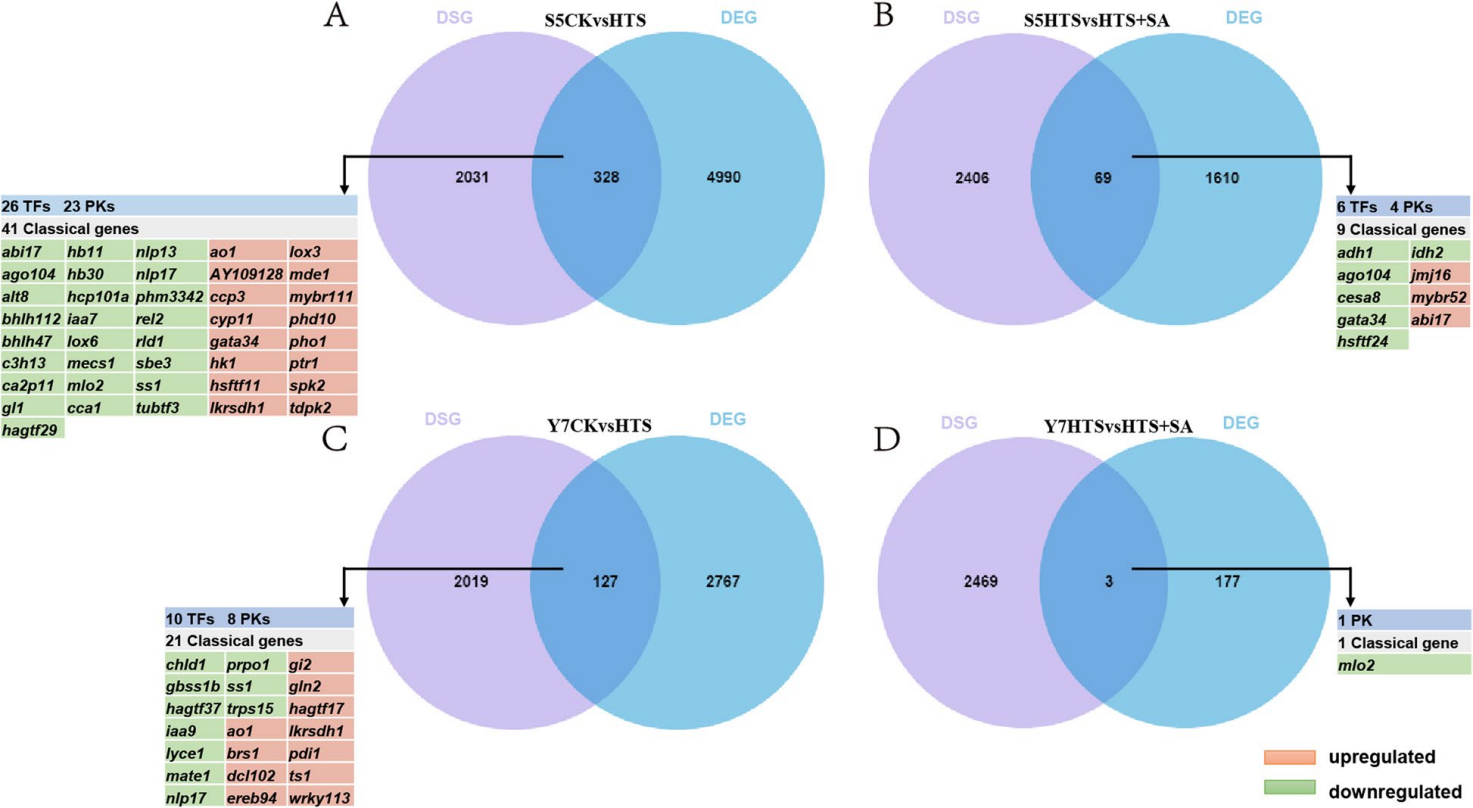
Venn diagrams showing the overlapping genes between DEGs and DSGs. **A**, S5CK vs HTS comparison. **B**, S5HTS vs HTS + SA comparison. C, Y7CK vs HTS comparison. D, Y7HTS vs HTS + SA comparison. The TFs, PKs, and classical maize genes among DSGs-DEGs overlap genes are listed. In the classical maize genes list, red represents upregulated and green indicated downregulated

In order to elucidate the relationship between AS and transcriptional regulation, the genes undergoing DAS with transcriptional changes in response to HTS and SA were compared. When comparing of DEGs to DSGs, 328 and 69 overlapping genes were found in S5CK vs HTS and S5HTS vs HTS + SA, respectively (Figs. 6 A and B). Additionally, 127 and 3 overlapping genes were found in Y7CK vs HTS and Y7HTS vs HTS + SA, respectively (Figs. 6 C and D). The majority of DSGs across the four groups were specific genes, suggesting that these genes were primarily regulated by AS under HTS and SA treatments. Of these overlapping genes, 26 TFs, 23 PKs, and 41 experimentally validated function maize genes (“classical maize genes” [37, 38]) were identified in S5CK vs HTS, while 6 TFs, 4 PKs, and 9 classical genes were identified in S5HTS vs HTS + SA. In addition, 10 TFs, 8 PKs, and 21 classical genes were identified in Y7CK vs HTS, and 1 TF and 1 classical gene were identified in Y7HTS vs HTS + SA.

## Discussion

Under the threat of climate change, improving the heat tolerance of maize is a particularly important goal in maize breeding. Studies have consistently shown that HTS is significantly damaging to the health, growth, and biomass accumulation of maize [39, 40]. Maize subjected to HTS, both MDA and ROS are overproduced and accumulate in organelles, thereby causing oxidative damage [5]. In response, plants activate a series of antioxidant enzymes to scavenge ROS [41]. In this work, we found that the increased accumulation of MDA in response to HTS led to the inhibition of SOD, POD and CAT, and that the effect of HTS on S5 was more severe than on Y7 (Fig. 1). SA has been found to have a broad range of functions related to plant abiotic stress response [14, 42]. Exogenous application of SA has been reported to both modulate the antioxidant defense system and mitigate HTS damage in plants [13, 17]. Regardless of concentration, exogenous SA pretreatment enhanced the activities of antioxidant enzymes, decreased the content of MDA, and increased biomass accumulation in waxy maize subjected to HTS (Fig. 1). Notably, the optimum SA concentration was different between S5 and Y7 seedings: 0.55 and 0.75 mM SA, respectively. This is in accordance with other studies which indicate that specific dosage is important to effectively modulate plant tolerance to stress [14].

Changes in endogenous phytohormone balance can directly impact heat tolerance in plants [43]. HTS decreased the concentration of endogenous ABA, IAA, SA, and GA3 in S5, but not in Y7, suggesting that Y7 is more thermotolerant (Fig. 1). These phytohormones have been reported to be positive regulators of heat tolerance [13, 44, 45]. Under HTS, phytohormones modulate the activities of antioxidant enzymes by interacting with ROS signaling, thereby alleviating oxidative damage in plants [46]. The exogenous application of these phytohormones, including SA, has been found to increase thermotolerance in plants [47]. SA, specifically, regulates plant abiotic stress response through feedback with other endogenous hormones [23]. Changes in phytohormone homeostasis therefore regulate and mediate complex stress-adaptive signaling pathways [48, 49]. Regardless of concentration, exogenous SA pretreatment significantly increased the content of ABA, SA and GA3 in both varieties. In the transcriptome, we found that S5 had a greater number of DEGs in plant hormone signal transduction pathway than Y7 (Fig. 3). Further, between CK and HTS, downregulated DEGs tended to be enriched in IAA and SA signal transduction pathways, mirroring the decrease in these hormones under HTS. In contrast, most DEGs related to the GA3 and ABA pathways were upregulated by HTS. HTS is known to induce a transient increase in the ABA and GA3 content of plants, both of which modulate the upregulation of downstream genes to reduce stress damage [50, 51]. Pretreatment with SA tended to reverse the expression of plant hormone signal transduction pathway genes, although more so in S5 than in Y7 (Fig. 3). These results indicate that SA interacts with the biosynthesis and signal transduction of other phytohormoes, improving heat tolerance in heat-sensitive waxy maize.

To protect themselves from severe HTS-induced damage, plant cells require HSFs for the rapid transcriptional activation of HSPs [52]. HSPs are molecular chaperones involved in repairing and refolding damaged proteins [53]. Thus, plant maintain homeostasis under HTS by extensively upregulating the expression of both HSFs and HSPs [54]. Phytohormones further enhance heat tolerance by mediating the expression of HSPs [47]. HSPs, in turn, enhance heat tolerance by scavenging ROS, maintaining membrane stability, and positively regulating the antioxidant enzyme system [55]. In the present study, 12 HSFs and 14 HSPs in S5 and 4 HSFs and 14 HSPs in Y7 were activated under HTS (Fig. 4B). Notably, SA pretreatment led to the downregulation of the 12 HSFs and 14 HSPs in S5, implying that SA was able to alleviate HTS in this variety. Previous study haves found that HSPs also play an important role in protecting the photosynthetic machinery from HTS-induced damage [56]. The photosynthetic machinery takes the brunt of the damage caused by HTS, making the photosynthetic system highly sensitive to HTS [57]. HTS primarily affects photosynthesis and the expression of photosynthesis-related genes by increasing the generation of ROS, destroying photosystem II [40, 58]. In this work, HTS induced the downregulation of photosynthesis-related genes in both varieties (Fig. 4A). Further, SA pretreatment was found to induce the upregulated of 5 photosynthesis-related genes in S5 exposed to HTS. This is similar to study in tomato, which indicate the exogenous SA enhances both photosynthesis efficiency and heat tolerance by scavenging ROS [17].

AS is a central post-transcriptional regulatory mechanism which works to address abiotic stress in plants [28]. Further, several studies have demonstrated that environmental stress significantly alters AS dynamics in plants [31, 59]. In this study, both HTS and HTS + SA treatments increased the number of AS events in both varieties (Fig. 5), which is consistent with previous studies in other plants exposed to environmental stress [60, 61]. Compared with HTS, SA pretreatment decreased the number of AS events in S5, but increased the number of AS events in Y7, suggesting that SA increased thermotolerance in heat-sensitive seedlings. Meanwhile, 2359, 2475, 2146 and 2472 DSGs underwent DAS in S5CK vs HTS, S5HTS vs HTS + SA, Y7CK vs HTS, and Y7HTS vs HTS + SA, respectively (Fig. 6). Notably, we found little overlap between DSGs and DEGs, leading to large differences in GO and KEGG enrichment between DSGs and DEGs (Fig.S2). These results suggest that AS and transcription regulation function independently under HTS, which is the case for *Arabidopsis* [62], tea [31], and rice [63].

Among the DEGs-DSGs overlap genes, several TFs and maize classical genes play important roles in regulating plant HTS response were identified. Previous work has shown that AS events may modulate stress response by targeting the ABA pathway [28]. We found several ABA-related genes which underwent AS in response to HTS, including *abi17* (Zm00001d003601) and *iaa7* (Zm00001d039513) in S5CK vs HTS, and *iaa9* (Zm00001d040541) in Y7CK vs HTS. In hybrid maize, HTS adaptation is strongly correlated with the induction of AS events in HSP genes [64]. We found 2 HSFs with AS transcripts were downregulated in S5CK vs HTS and upregulated in S5HTS vs HTS + SA. Certain genes may affect resistance to multiple abiotic stresses, such as *ZmCCA1* (*CIRCADIAN CLOCK-ASSOCIATED 1*), the overexpression of which leads to enhanced drought tolerance in *Arabidopsis* [65]. *ZmCCA1* was downregulated by HTS in S5, suggesting this gene may also affect heat tolerance in maize. Furthermore, the starch synthesis pathway genes *sbe3*, *ss1*, and *gbss1b* were downregulated by HTS in both varieties. In transgenic wheat, the overexpression of rice *SSI* gene has been found to improve photosynthetic efficiency and heat tolerance [66]. Collectively, our results suggest that transcriptional regulation and AS may function concertedly in waxy maize leaves to enhance tolerance to HTS.

## Conclusions

**Fig. 7:**
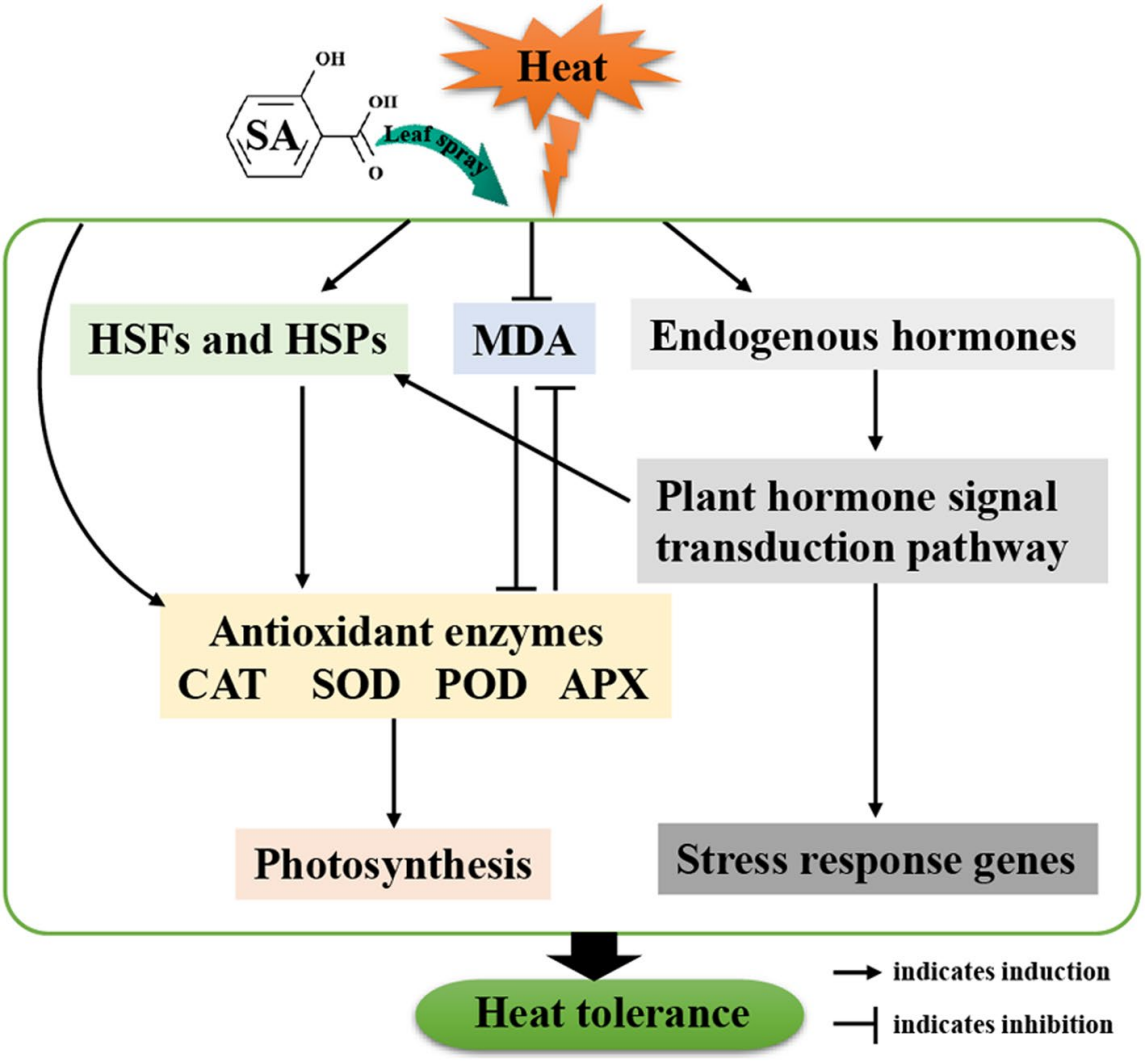
A descriptive model of SA enhancing heat tolerance of waxy maize seedling under heat stress

In summary, our results suggest that exogenous application of SA works to ameliorate HTS-induced damage, particularly in heat-sensitive waxy maize variety. We propose a simplified model to describe the role of exogenous SA in heat tolerance (Fig. 7). Specifically, SA works to reduce the content of MDA and increase the activities of antioxidant enzymes and the concentrations of phytohormones. Furthermore, exogenous application of SA downregulated the expression of genes encoding HSFs and HSPs, and upregulated the expression of genes involved in photosynthesis, suggesting that SA works to both increase photosynthetic efficiency and heat tolerance. It appears that waxy maize may have specific responses at the transcriptional and AS levels to adapt to HTS. Our findings provide valuable information to understand the biological roles of SA in waxy maize heat tolerance and its application in future production.

## Methods

### Plant materials, growth conditions, SA and heat treatments

The waxy maize varieties of Suyunuo5 (S5, heat-sensitive) and Yunuo7 (Y5, heat-tolerant) were selected as materials. They were sown in 7 cm (diameter) × 8 cm (height) plastic pots with the 50% (v/v) peat-based soil and 50% (v/v) vermiculite. Plants were grown in a light incubator at 28 °C (light, 14 h) /23 °C (dark, 10 h) and a relative humidity of 60% until the three-leaf stage [67]. Next, some maize plants were sprayed with different concentrations of SA: 0.35, 0.55, and 0.75 mM. Moreover, 0.05% (v/v) Tween-20 was used as a surfactant. After 12 h of treatment, these plants were given heat at 42 °C (light, 14 h) /37 °C (dark, 10 h) for 48 h. The experiment included five treatments as follows: 1) CK (control, sprayed with water without heat), 2) HTS (HTS treatment, sprayed with water), 3) HTS + 35SA (0.35 mM SA pretreatment before HTS treatment), 4) HTS + 55SA (0.55 mM SA pretreatment before HTS treatment), 5) HTS + 75SA (0.75 mM SA pretreatment before HTS treatment). Each treatment group had 30 pots. Half-strength Hoagland nutrient solution was applied every 2 days. After HTS treatment, all leaves of three seedlings under the same treatment were collected and pooled as one replication. Leaf samples from each treatment were stored at − 80 °C freezer for physiological indicators and transcriptome analyses.

### Measurement of physiological characterization

The shoot dry weight weighted after the aboveground parts of plants was dried in an oven at 105 °C for 30 min and then held at 80 °C for 12 h. The frozen leaf samples were used to measure the contents of MDA, ABA, IAA, GA3, and SA, and the activities of SOD, POD, APX, and CAT. All measurements were performed using the kits produced Shanghai Enzyme-linked Biotechnology Co., Ltd. (Shanghai, China) and performed four biological replicates. The concentrations of MDA, ABA, IAA, GA3, SA, SOD, POD, APX, and CAT in the leaf samples were then determined based on the Optical Density of the samples and the standard curve [68, 69].

### RNA extraction, library construction, and sequencing data analysis

Based on the physiological characterization results, we selected leaf samples under CK and HTS treatments of two varieties (labeled as S5CK, S5HTS, Y7CK, and Y7HTS), and HTS + 55SA treatment of S5 (labeled as S5HTS + SA), and HTS + 75SA treatment of Y7 (labeled as Y7HTS + SA) for transcriptome analysis. Total RNA was extracted from 18 leaf samples (with three biological replicates) using the TRIzol reagent (Tiangen, Shanghai, China). RNA sequencing (RNA-seq) was performed on the Illumina NovaSeq™ 6000 by Biomarker Technologies Corporation (Beijing, China), and 150-base paired-end reads were generated. The raw reads were processed by BMKCloud (www.biocloud.net) online platform. The quality information of the RNA-seq was listed in Table S1. The sequencing data were deposited in the NCBI SRA database (BioProject ID: PRJNA842821). Clean reads were aligned to the B73 reference genome Gen_v4 (download from the website: ftp://ftp.ensemblgenomes.org/pub/release-32/plants/fasta/zea_mays/dna) using HISAT2 v2.0.4 [70]. The expression abundances of mapped reads were counted and normalized into fragment per kilobase of transcript per million mapped reads (FPKM) method using StringTie v1.3.4d [71]. Differentially expressed genes (DEGs) were performed by DESeq2 v1.6.3 and screened by following criterion: |log_2_ fold change| ≥ 1 and *p*-value < 0.05.

### Alternative splicing (AS) analysis

After reads mapping and transcript assembling, AS events were identified and classified by ASprofile [72]. Differentially alternative splicing (DAS) events of skipped exon (SE), alternative 5′ splice site (A5SS), alternative 3′ splice site (A3SS), mutually exclusive exons (MXE) and retained intron (RI) were analyzed by rMATS v4.0.2 and screened by FDR < 0.01 [73]. Genes involved in DAS events were consider as differentially spliced genes (DSGs).

### Functional enrichment analysis

Gene Ontology (GO) of DEGs and DAS genes were carried out by GOseq R software package [74]. GO terms with corrected *p*-value (*q*-value) < 0.05 were considered significantly enriched terms. Kyoto Encyclopedia of Genes and Genomics (KEGG) pathway annotation and enrichment analysis of DEGs and DAS genes were performed using KOBAS software [75], with a corrected *p*-value (*q*-value) < 0.05 as the threshold for significantly KEGG pathways.

### Quantitative real-time-PCR analysis

To further validate the expression profiles of genes in RNA-seq, eight DEGs were randomly selected for qRT-PCR validation. The ChamQ Universal SYBR qPCR Master Mix (Vazyme, Nanjing, China) was used for qRT-PCR on a Real-time fluorescent quantitative PCR instrument ABI ViiA™ 7 (Applied Biosystems, CA, USA). The relative expression levels of the genes were calculated using the 2 ^−∆∆Ct^ method, and *GAPDH* was used as an internal control (reference gene) [76]. The qRT-PCR primers sequences were designed using Primer Premier 6.0 software (Premier Biosoft International, Palo Alto, CA, USA) and listed in Table S3.

### Statistical analyses

Statistical analysis were performed using SPSS v. 19.0 (IBM, Armonk, NY, USA), and Duncan’s multiple range test was performed with a significant difference set at *P* < 0.05. Histograms were made by GraphPad Prism software v.8.0.0 (San Diego, CA, USA). Heatmaps were made by TBtools software v.1.098745.

## Supplementary Information

**Additional file 1 Table S1.** Summary of 18 RNA-seq samples with QC quality.

**Additional file 3 Table S2.** List of plant hormone signal transduction pathway related DEGs in different comparisons.

**Additional file 2 Table S3.** Primer sequences of DEGs encoding genes used for qRT-PCR.

**Additional file 4 Fig. S1.** Transcriptomic data, gene expression levels and principal components analysis. A, Heatmaps showing the correlations between the transcriptomes of three biological replicates under different treatments of two varieties. B, Gene expression levels under different treatments of two varieties. C and D represent the principal components analysis of different treatments for S5 and Y7, respectively.

**Additional file 5 Fig. S2.** GO terms enrichment and KEGG pathway analysis of DSGs in CK vs HTS and HTS vs HTS + SA comparisons of two varieties.

**Additional file 6 Fig. S3.** mRNA expression level analysis (qRT-PCR) of eight DEGs. The Log_2_ (fold change) value represented the CK vs HTS and HTS vs HTS + SA comparisons for two varieties.

## Data Availability

The transcriptome datasets supporting the conclusions of this study are available in the NCBI (BioProject: PRJNA842821).
