# Supplementary figures and images for: Transcriptomic and alternative splicing analyses provide insights into the roles of exogenous salicylic acid ameliorating waxy maize seedling growth under heat stress
Source: BMC Plant Biol. 2022 Sep 9;22:432. doi: 10.1186/s12870-022-03822-3 (PMC9461148; doi:10.1186/s12870-022-03822-3)

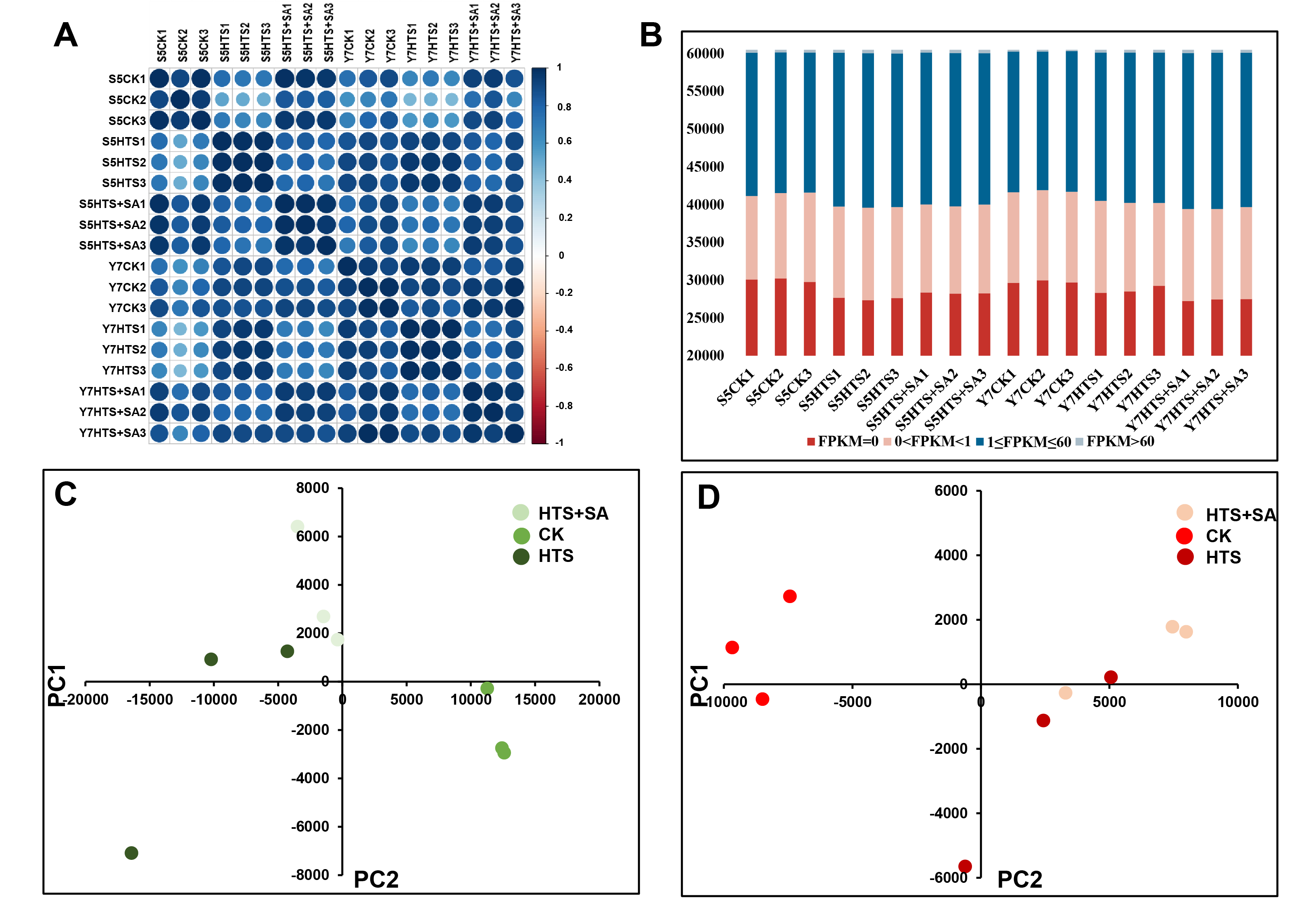

Supplement: Supplementary file 4 — Additional file 4 Fig. S1. Transcriptomic data, gene expression levels and principal components analysis. A, Heatmaps showing the correlations between the transcriptomes of three biological replicates under different treatments of two varieties. B, Gene expression levels under different treatments of two varieties. C and D represent the principal components analysis of different treatments for S5 and Y7, respectively. [file 12870_2022_3822_MOESM4_ESM.tif]

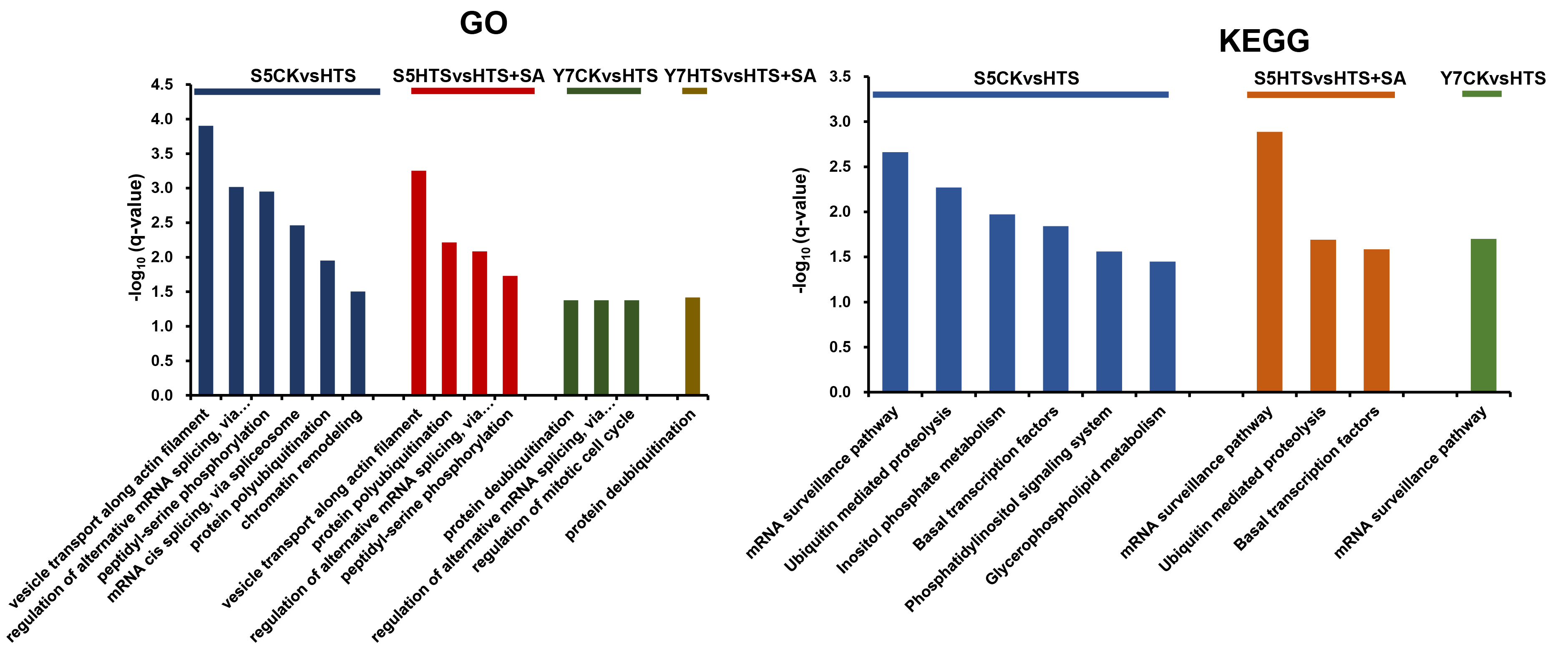

Supplement: Supplementary file 5 — Additional file 5 Fig. S2. GO terms enrichment and KEGG pathway analysis of DSGs in CK vs HTS and HTS vs HTS + SA comparisons of two varieties. [file 12870_2022_3822_MOESM5_ESM.tif]

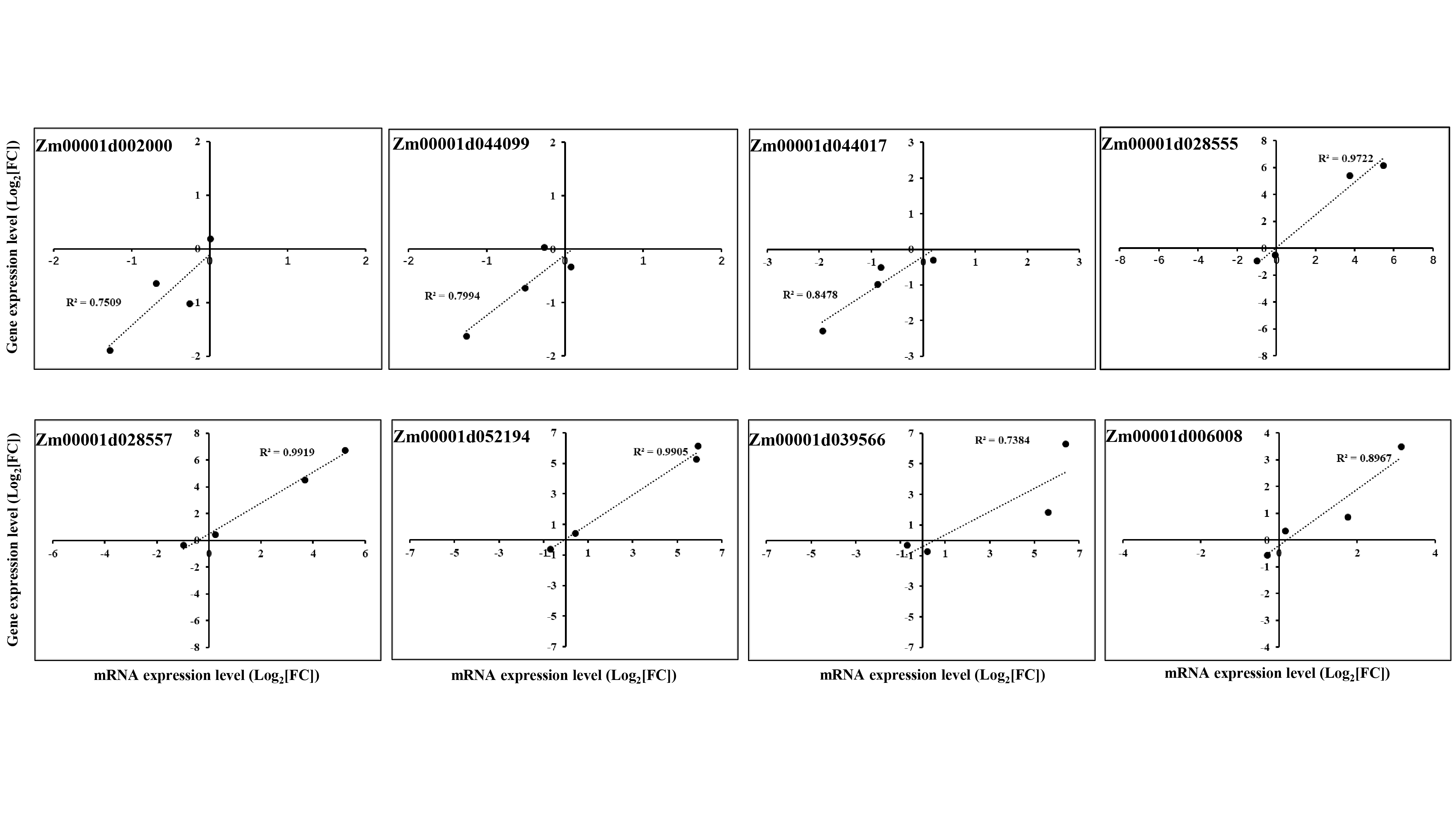

Supplement: Supplementary file 6 — Additional file 6 Fig. S3. mRNA expression level analysis (qRT-PCR) of eight DEGs. The Log2 (fold change) value represented the CK vs HTS and HTS vs HTS + SA comparisons for two varieties. [file 12870_2022_3822_MOESM6_ESM.tif]
